# Principles of Comparative Physiology

**Published:** 1854-10

**Authors:** 


ART. V.—*Principles of Comparative Physiology.* By WILLIAM B. CARPENTER, M. D., F. R. S., F. G. S. &c., &c., &c. A new American from the fourth and revised English edition. Philadelphia: Blanchard & Lea. 1854.

*The Principles of Animal and Vegetable Physiology; a popular treatise on the functions and phenomena of organic life. To which is prefixed a general view of the great departments of human knowledge.* By J. STEVENSON BUSHNAN, M. D., etc. Philadelphia: Blanchard & Lea. 1854.

*The Principal Forms of the Skeleton and Teeth.* By Prof. R. OWEN, M. D., F. R. S., etc. Philadelphia: Blanchard & Lea. 1854.

Carpenter's Comparative Physiology is already widely known to the American medical public through previous editions. Of its merits as the most comprehensive of all accessible treatises on this subject it is unnecessary for us to speak.

The two accompanying works are republications from a series of treatises now publishing in London, under the general title of "Orr's Circle of the Sciences." Dr. Bushnan is the editor of the series, and with him are associated the names of Owen, Ansted, Latham, and others. If we are to judge of the promise of the series from these names, and the volumes here under review, we have reason to be thankful to Messrs. Blanchard & Lea for this last instance of that enterprise, which has already made them pre-eminently the medical book publishers of this country.

In no one of the circle of sciences has there been that rapid and manifest advancement, which has recently characterized the progress of physiological research. For a long period the science of physiology rested on that knowledge only which might be inferred from the ocular inspection of the human body, and from a comparatively barren human anatomy. But of late years the revelations of the miscroscope, and the careful study of comparative anatomy down through the scale of descent to the lowest invertebrata, has opened a wide avenue of progress. Anatomy itself, that science which was supposed to have few problems left for the student, is widening before us, a vast and fruitful field. The mystery of organic life increases as we simplify its phenomena, and the mind is startled as it finds that an explanation of the hitherto incomprehensible but entails upon it another and more difficult problem.

Accustomed to the study of the highest form of organization, commencing with the complex and abstruse, the student finds in anatomy but a tedious web of words, a wearisome nomenclature, and suffers constantly from a feeling of deficiency, a knowledge that some intrinsic elements are omitted. Few become enthusiasts in anatomy as taught in our schools, for the plain reason that they are studying algebra before arithmetic, philology before grammar, the complex before the simple, the highest forms of life before they have learned the alphabet of simple organizations.

The remedy for this is to begin at the beginning, to trace the early conceptions of formative Power, the first attempts at organization. For it is a fact no less strange than beautiful, that out of the simplicity of primary forms, the rude shapes of a primeval age, has grown perfection; its progressive ages being marked in the rocks which perpetuate the forms of extinct races. And in still living fishes and insects we may discover how much of organism is necessary to existence; in a word, what, and how little, are the essential parts which make the living being. Here, too, are found the archetypes of higher life; the rude sketch, the imperfect conception, which, elaborated and perfected in the human race, excites our admiration. Thus, reasoning down the chain, we reach at last the typical form of every tissue, the model on which all more complex forms are built. By its aid the mind is relieved of all the endless detail which perplexes the novice, and strips from the most complicated form all that is non-essential to the type. The mind transcends the senses, we have an anatomy not demonstrable to the eye, but clearly visible to the reason. Such is transcendental, or (to use a better word) Philosophical Anatomy.

Says Oken, "It is truly remarkable what it costs to solve any one problem in philosophical anatomy. Without knowing the *what*, the *how*, and the *why*, or to what end they are modified, one may stand, not for hours or days, but weeks, before a fish's skull, and our contemplation will be little more than a vacant stare at its complex, stalactitic form."

In no way can the history of the cell be so well taught as by reference to the simple sporules of the cryptogamia. Here, piled up one upon another, rest these autocthynes of cell-life; the simple forms upon the different aggregation of which depend the myriad shapes of organism. The same holds true of the harder tissues, in which a larger proportion of inorganic matter enters.

As an illustration, we draw from Owen the following ideas on the philosophical anatomy of the skeleton.

In the four classes which make up the highest province of the animal kingdom — the vertebrata — the generic name is of course derived from the fact that all these classes, whether Fishes, Reptiles, Birds, or Mammals, possess a series of confluent bones, on which the body turns. The simplest form of the vertebra, or rather its typical form in which it possesses only its essential features, is represented by a mass of bony matter called the centrum, from which spring two arches, one above, and one below. The upper of these is designed for the protection of the nervous tissues, and is accordingly styled the "neural arch;" while the lower, inclosing more or less perfectly within it the blood making viscera, is called the "hæmal arch." The various pieces which make up these several arches derive names expressive of their philosophical use from the organs they protect. Thus the hoop which protects the spinal marrow is formed by two bones called "neurapophyses," while another fragment, interposed between their extremities, and which is sometimes bifid, is called the "neural spine." Applying this nomenclature to a dorsal vertebra, we have its body representing the "centrum," its arch inclosing the spinal marrow as a "neurapophysis," and its spinous process as the "neural spine."

The "hæmal arch" is a hoop anterior to this, formed by two bones called "pleurapophysis," *(pleuron*, rib,) by a second pair called "hæmapophysis," *(haima*, blood,) and, finally, a central bone called the "hæmal spine."

Pursuing our comparison, we have the rib articulating with the centrum as the "pleurapophysis," the cartilages (undeveloped bone) as the "hæmapophysis," and the sternum as the "hæmal spine." All other parts attached to these are not what the author calls "autogenous," that is, intrinsic; but "exogenous," that is, unnecessary to the essentiality of the organism. The sacrum is to be considered as a series of connate vertebræ, the hæmal arch being formed by the pelvic bones. The upper and lower extremities are not parts of the organism proper, but only means of locomotion for carrying it about, or for prehension of food. Like the fins of a fish, they are rays of ossification, extending from the vertebræ.

The cranium is made up of four vertebræ, the true cranial bones representing the different neural arches, while the facial bones proper make up the hæmal arches.

It is less difficult to consider the cranium as a series of confluent vertebræ, than to extend the idea to the trunk. In very many of the lower animals, especially some long billed birds, the foramen magnum, and occipital bones present a resemblance to a vertebra which strikes the most unaccustomed eye.

It is argued that all these changes from the typical vertebra are merely adaptations to special uses, the conforming of the casket to the convenience of its contents. So wide, however, are the departures from the type, that it is by no means easy to trace the original form, or detect the original purpose. But from such studies, however fanciful they may appear to the unfamiliar eye, are evolved the highest truths of anatomy and physiology. It is not necessary that in order to profit by them we should adopt the full extent of the theory, and whoever will carefully consider the resemblances alledged, will find that, though he may refuse assent to many of the details, he must still adopt the basis of the doctrine.

The works mentioned above may be procured from Messrs. Miller, Orton & Mulligan. H.

